# Evaluation of totally implantable catheters in healthy horses

**DOI:** 10.1186/s12917-021-03052-z

**Published:** 2021-10-26

**Authors:** Adriana Fernandes de Souza Garcia, Gesiane Ribeiro, Julia de Assis Arantes, Gustavo Morandini Reginato, Nathalia Villaca Xavier, Adriano Bonfim Carregaro, Thiago Jhonatha Fernandes Silva, Renan Grigoletto, Silvio Henrique de Freitas, Renata Gebara Sampaio Dória

**Affiliations:** 1 United Metropolitan Colleges, Rua Ministro Nelson Hungria, 541, Vila Tramontano, São Paulo, São Paulo 05690-050 Brazil; 2 Department of Veterinary Medicine, Faculty of Animal Science and Food Engineering, University of São Paulo, Rua Duque de Caxias Norte, 225, Jardim Elite, 13.635-900, Pirassununga, São Paulo, Brazil

**Keywords:** Complications, Equine, Implantation technique, Intravenous regional limb perfusion, Long-term catheter

## Abstract

**Background:**

For horses requiring prolonged daily cephalic intravenous regional limb perfusion (IVRLP), the use of a totally implantable catheter (TIC) could be indicated to reduce complications associated with frequent venipuncture or external catheterization. This study aims to evaluate the implantation technique of the TIC in the cephalic vein of horses for IVRLP, describe the complications associated with the device’s placement and use, and assess its viability up to 60 days after implantation. Totally implantable catheters, cut to 15 cm (*n* = 5) and 46 cm (n = 5) in length, were implanted into one cephalic vein in ten adult horses (*n* = 10). Twenty-four hours following placement, IVRLP with contrast was performed via the TIC and evaluated with radiography. Physical examinations, lameness evaluation, hematologic assessment, and the catheter patency tests were performed at scheduled intervals for the duration of catheterization (7–60 days).

**Results:**

Catheters were implanted without difficulty and allowed for IVRLP 24 h post implantation. Complications resulted in removal of the catheters, with four maintained for 7 days, three in place for 15 days, and three catheters maintained for 60 days. Complications included lameness, limb swelling, catheter kinking, and venous thrombosis.

**Conclusions:**

The implantation technique of the TIC in the cephalic vein of horses is feasible and requires minimal technical effort. Although TIC allows venous access without the need for repeated venipuncture, its long-term use presents complications. For horses requiring prolonged daily cephalic IVRLP, the use of a TIC could be indicated. However, the high incidence of venous thrombosis may limit clinical application.

## Background

Traditionally, intravenous regional limb perfusion (IVRLP) in horses is performed by venipuncture without a catheter or using short-term peripheral catheters [1–3], which are often only inserted at the time of the procedure [4–6]. In many clinical cases, repeated IVRLP is necessary to resolve persistent orthopedic infections [7–10]. However, repeated venipuncture can injure the regional vein, making vessel handling difficult and favoring perfusate leakage [1–3]. In this case, premature discontinuation of IVRLP treatment usually occurs due to thrombophlebitis or loss of venous access [8–10]. To avoid daily venipuncture and to facilitate perfusion, a long-term indwelling catheter, over-the-needle or over-the-wire, can be placed in a regional vein and remain patent for 7 days, although complications including local inflammation, lameness, and thrombophlebitis have been reported [11, 12].

Long-term catheters are classified as either external, semi-implantable catheters, or totally implantable catheters (TICs) [6]. Currently, semi-implantable and totally implantable catheter made of silicone or polyurethane, of single or multiple lumens, are used in human patients for hemodialysis, hemotherapy, chemotherapy, and prolonged parenteral nutrition [13]. TICs are surgically implanted in large vessels and connected to an administration device (titanium reservoir) housed in a subcutaneous pocket. Medications can be administered as a bolus or by continuous rate infusion through this reservoir, which is accessed by puncturing the intact skin with a thin-caliber Huber needle [4, 5, 14]. Studies investigating the use of TICs in people reported venous access for 353 days on average, absence of complications in 82.2% of the cases, and a patient satisfaction rate of 93% [15, 16].

The objective of this study was to adapt and evaluate the feasibility of implanting TICs in the cephalic vein of horses and to evaluate the devices’ functionality, patency, and prolonged viability for use in IVRLP while identifying possible systemic and specific complications (swelling, pain and lameness). Considering the satisfactory results obtained with TIC in humans, it was hypothesized the majority of this device would stay patent/flush for 60 days in equine limbs, thus reducing complications associated with frequent venipunctures or external catheterization for repeated IVRLP.

## Results

**Fig. 1:**
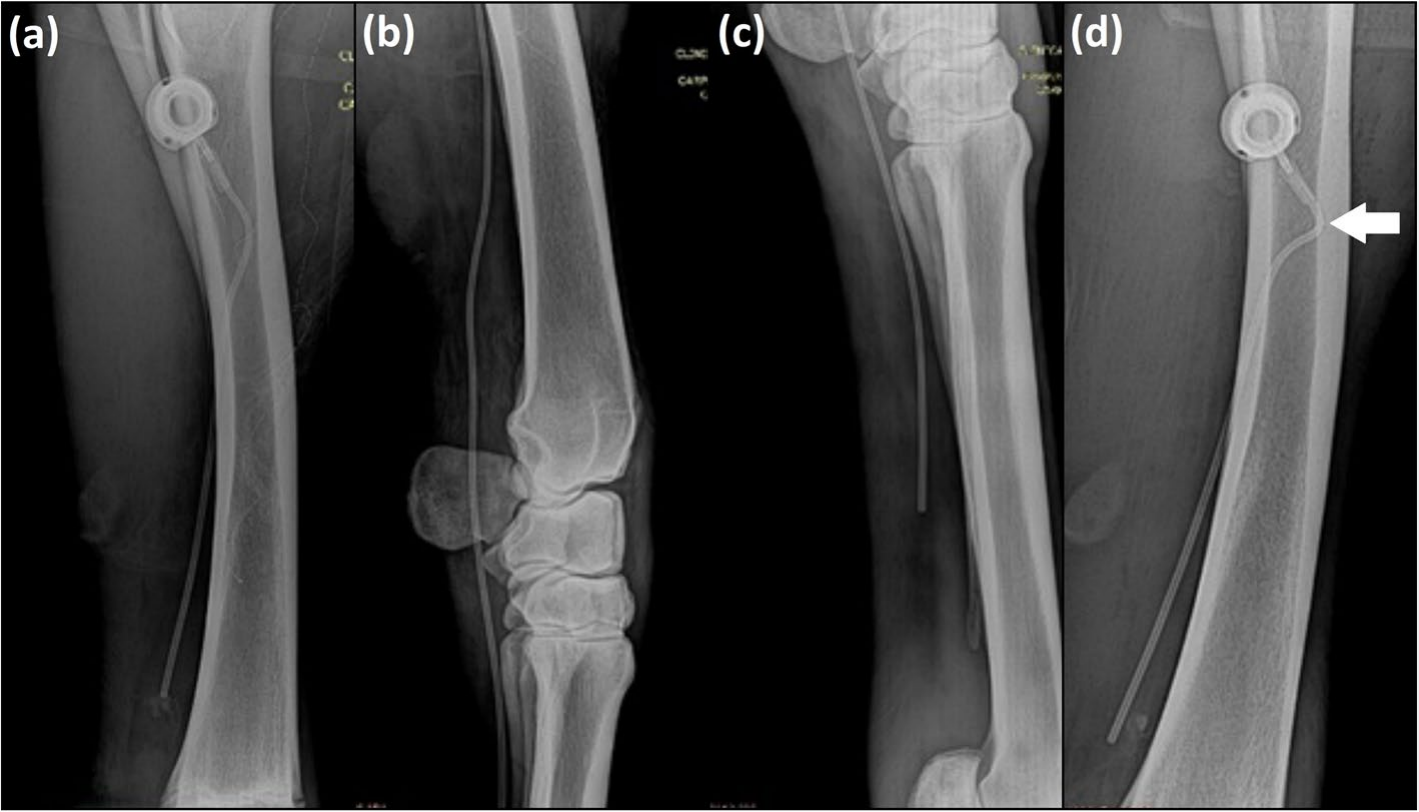
Radiographic images of the thoracic limbs of adult horses illustrating in (**a**): positioning of the 15-cm long totally implantable catheter, model Life-Port Titanium Infant 7.5 Fr in the cephalic vein during the peri-operative period of implantation. In (**b**): positioning of the 46-cm long totally implantable catheter model Life-Port Titanium Infant 7.5 Fr in the cephalic vein passing through the carpal joint and ending in the middle third of the third metacarpal bone (**c**). In (d): 24 h after the catheter implantation, standing position; the image shows a steeper curvature of the catheter at the site of coupling to the reservoir (arrow) and the more distal positioning of the reservoir relative to the ulna when compared to the same horse displayed in the image (**a**)

Implantation of the TIC in the cephalic vein of horses proved to be a feasible and a low difficulty procedure that took a mean time of 40 min (range 38–42 min) for completion. Lateral recumbency, general anesthesia, and radiographic monitoring were necessary for the catheters to be implanted aseptically and without technical errors. The 7.5-Fr catheter was compatible with the diameter of the cephalic vein of the study’ horses and allowed insertion of a total of 46 cm of the catheter within the cephalic vein, thus reaching distal portions of the limb. The 46-cm catheters passed distally over the region of the carpal joints and ended in the middle third of the metacarpus. The 15-cm catheters had their tips positioned in the distal third of the antebrachium, proximal to the carpal canal (Fig. 1A, B and C).

Based on perioperative radiographs, all of the TICs were correctly positioned within the cephalic vein, without kinking immediately following placement. Radiographic images obtained 24 h later with the horse standing again revealed correct positioning of the catheter although slight changes in the positioning of the reservoir and the curvature of the proximal aspect of the catheter were noted compared to the intraoperative images (Fig. 1D).

**Fig. 2:**
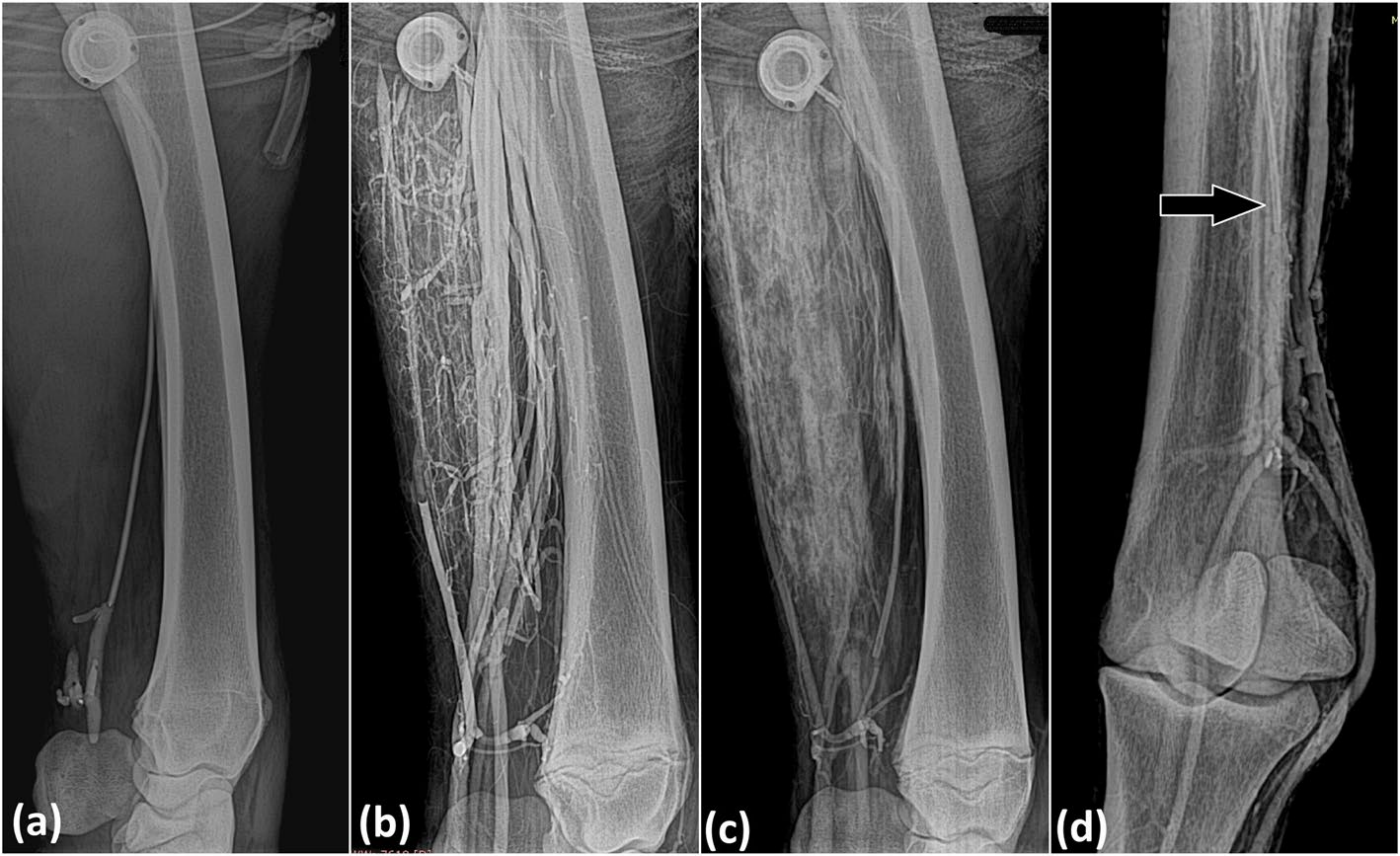
Radiographic images during cephalic IVRLP with contrast using a 15-cm long totally implantable catheter in a horse showing in (**a**) the infusion period and in (**b**) 10 min after contrast administration; (**c**) radiographic image of contrast diffusion through soft tissues after 30 min; (**d**) 46-cm long totally implantable catheter (arrow) in a horse, 10 min after contrast administration

The IVRLP with contrast solution was the strategy employed to show the use of the TIC. There were no technical difficulties in performing the IVRLP with the TIC. Radiographic assessment detected the flow of the contrast inside the vessels during the infusion and 10 min after contrast administration. After 30 min, the radiopacity of the tissues increased while the visualization of the vessels decreased (Fig. 2A, B, C and D).

**Fig. 3:**
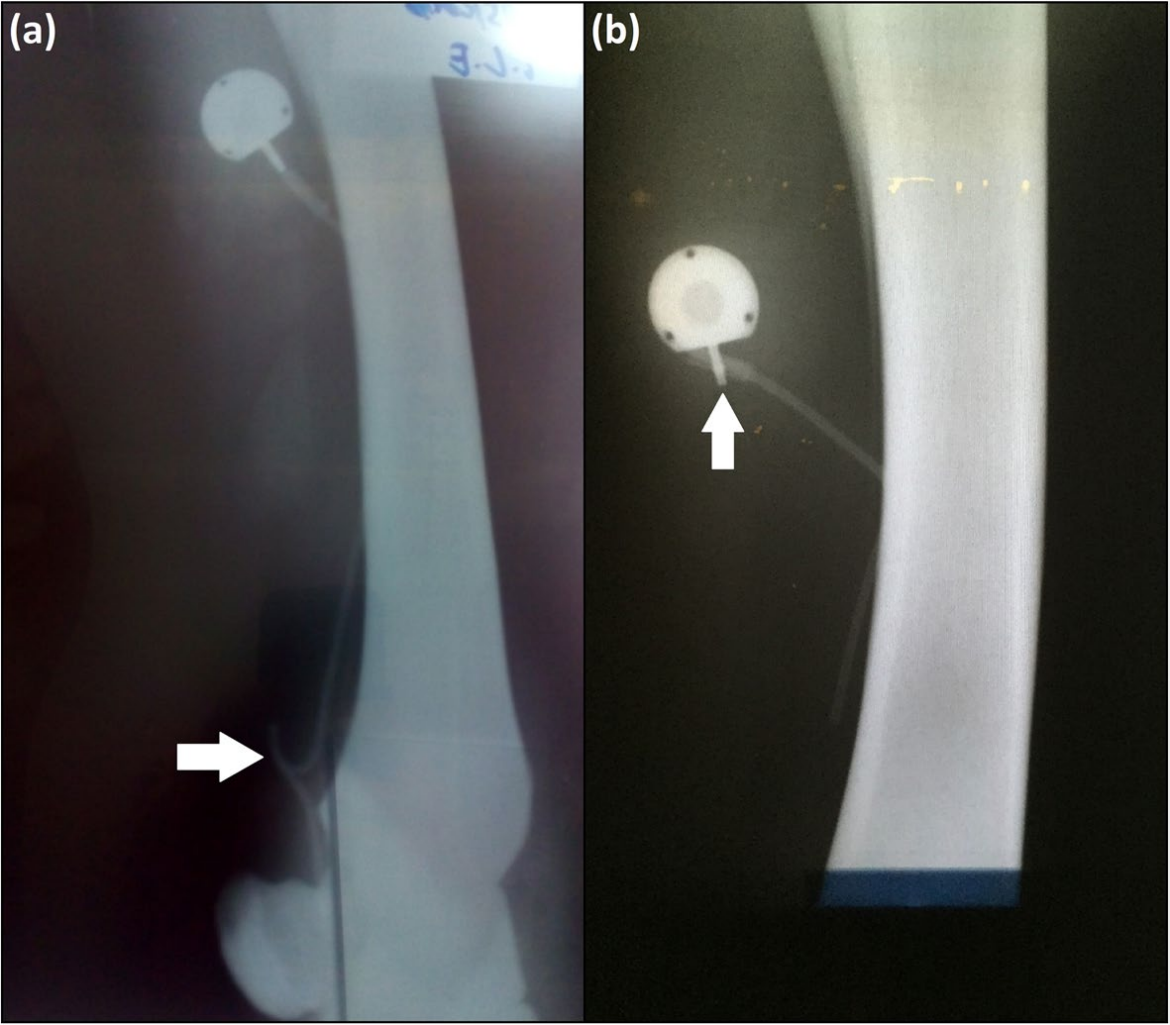
Radiographic images of the thoracic limbs of adult horses showing complications. **a**: 46-cm long totally implantable catheter with a kink (arrow) inside the cephalic vein. **b**: decoupling (arrow) of a 15-cm long totally implantable catheter

**Fig. 4:**
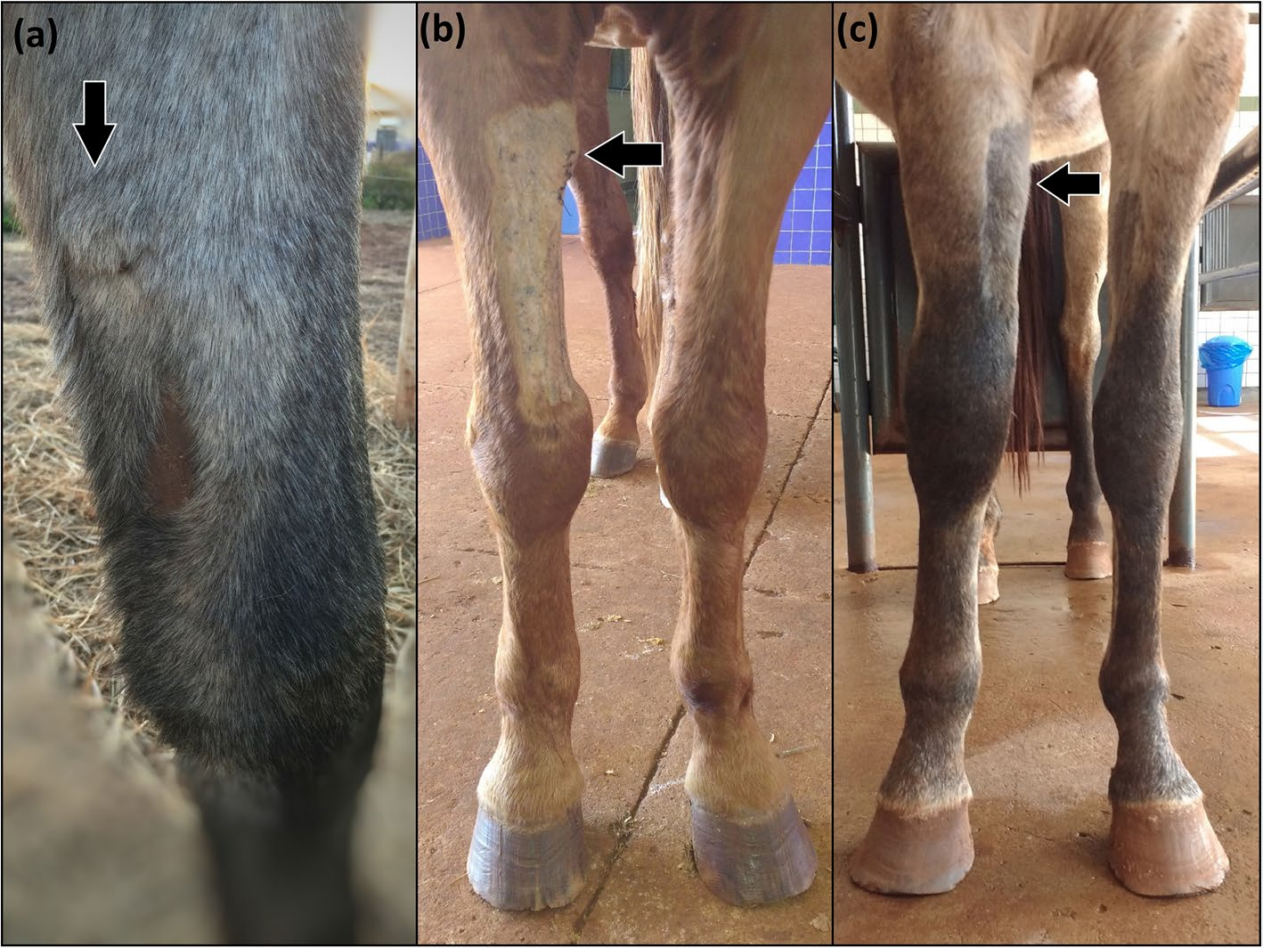
Image of the right thoracic limb of a horse after implantation of the totally implantable catheter in the cephalic vein. The arrows indicate the location of the reservoir. **a**: sixty days (Day 60); (**b**): seven days (Day 7), without limb swelling; (**c**): fifteen days (Day 15), note the presence of limb swelling

**Fig. 5:**
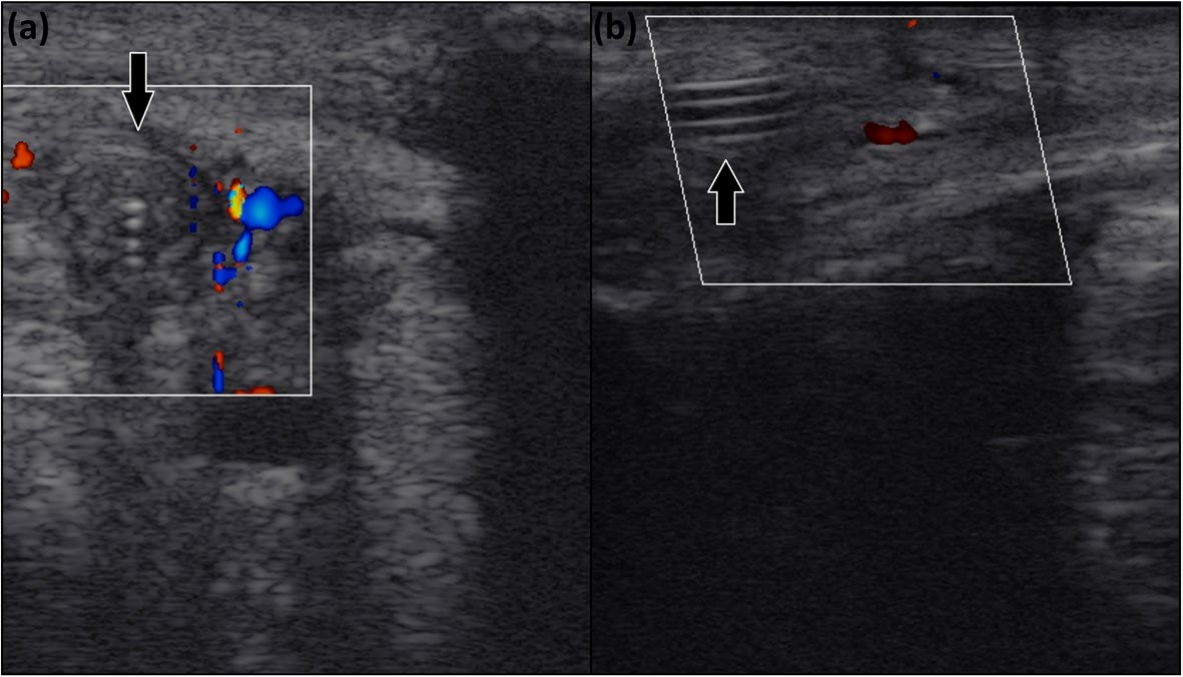
Ultrasound image obtained at cross-sectional (**a**) and longitudinal (**b**) views of the right thoracic limb of a horse in which the catheter (arrow) is detected inside the cephalic vein. Absence of blood flow is evidenced by the Doppler mode

The animals showed no changes over time and between groups in mucous membrane color (*p* = 1), capillary refill time (*p* = 1), heart (*p* = 0.977) and respiratory (*p* = 0.678) rates, intestinal motility (p = 1) and rectal temperature (*p* = 0.999). Skin sutures healed by first intention. Results regarding the catheter viability relative to the administration of fluids, decoupling, presence of kinking, edema and/or limb pain, the presence of venous stasis by ultrasonographic examination, and the time span until catheter removal are presented in Table 1 (more information is also shown in Fig. 3A, B, Fig. 4A, B, C, Fig. 5A and B).

**Table 1:** Totally implantable catheter viability relative to fluid administration (flushable or non-flushable) and complications as decoupling, presence of kinking, swelling and limb pain, venous stasis visualized at ultrasound and time at which the catheter was removed (catheter survival) in Groups G46 and G15

	Animal	flushable	non-flushable	decoupling	kink	swelling pain	venous stasis	catheter survival
**G46**	**G46A**	X		No	Yes	Yes	No	7 days
	**G46B**		X	Yes	No	No	No	60 days
	**G46C**	X		No	Yes	Yes	No	7 days
	**G46D**	X		No	No	No	No	60 days
	**G46E**	X		No	No	Yes	Yes	15 days
**G15**	**G15F**	X		No	No	Yes	Yes	7 days
	**G15G**	X		No	No	Yes	Yes	15 days
	**G15H**	X		No	No	Yes	Yes	15 days
	**G15I**	X		No	No	Yes	Yes	7 days
	**G15J**	X		No	No	No	No	60 days

Thirty percent of the catheters (*n* = 3) were maintained for 60 days, 30% (*n* = 3) for 15 days, and 40% (*n* = 4) for 7 days. Three horses (G46B, G46D, and G15J) did not show lameness, pain on palpation, swelling, and heat of the limb where the catheter was implanted, and therefore remained with the catheters in place for 60 days (Fig. 4A). At the time of catheter removal, the TICs were correctly positioned, except for one that presented decoupling (Fig. 3A), and no tissue reactions were detected.

**Fig. 6:**
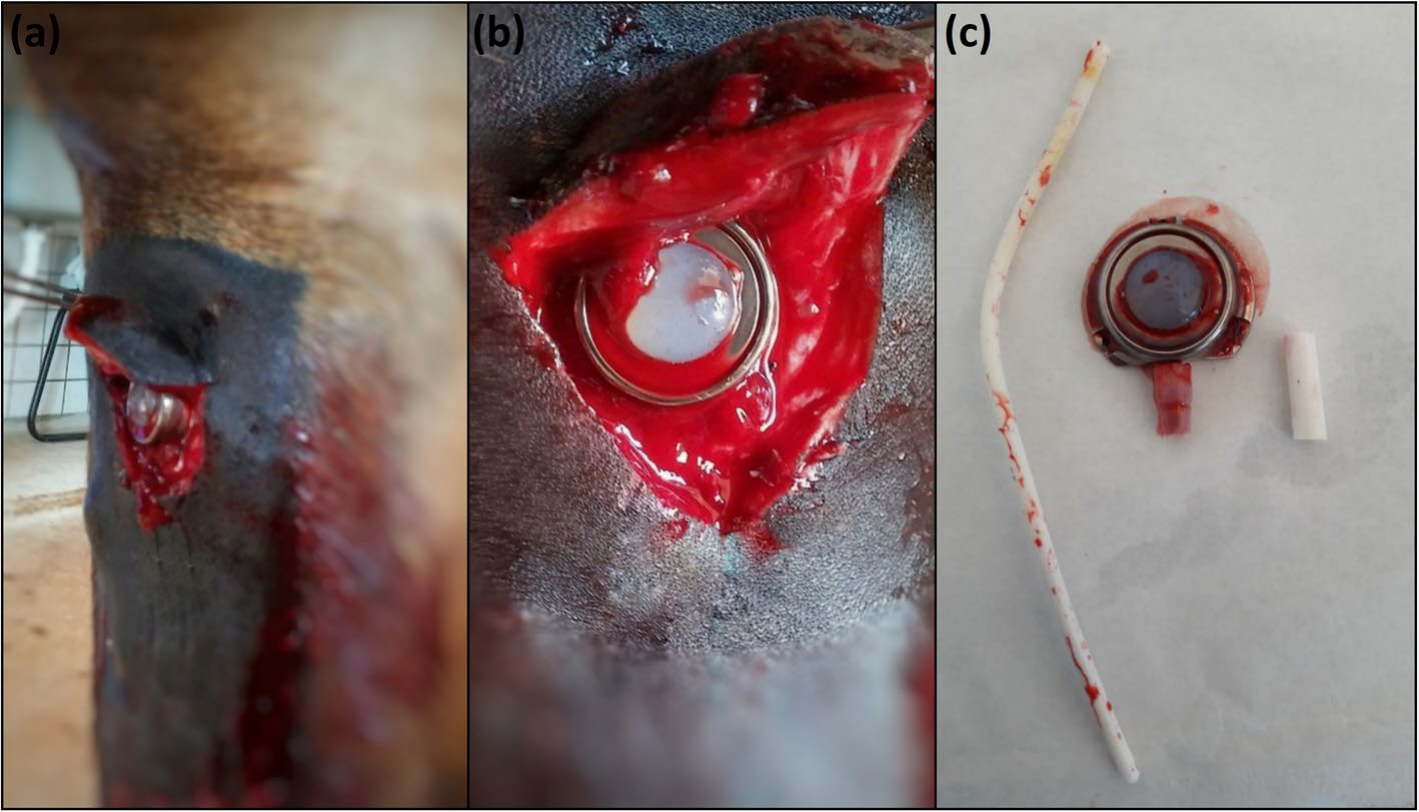
Images of the right thoracic limb of an adult horse showing in (**a**): removal of the totally implantable catheter; (**b**): note the absence of exudate or tissue necrosis; (**c**): totally implantable catheter removed

Seven catheters were removed before 60 days due to complications: four at 7 days (G46A, G46C, G15F, and G15I) and three at 15 days (G46E, G15G, and G15H). These seven presented with a grade 4/5 lameness, pain on palpation, swelling (Fig. 4C), and heat of the whole limb. Radiographic examination revealed catheter kinking in two horses (G46A and G46C; Fig. 3B). Ultrasonography indicated total venous occlusion evidenced by the absence of blood flow in the catheterized region of the cephalic vein, suggesting venous stasis and thrombosis in five horses (G46E, G15F, G15I, G15G and G15H; Fig. 5A and B). These catheters were immediately removed (Fig. 6A, B and C). Notably, the catheters were correctly positioned, without tissue reactions at the site where the reservoirs were placed. No purulent exudate or tissue necrosis was detected. No clotted blood was observed inside the catheters (Fig. 6C).

Compared to the baseline values (8.4 ± 1.7 × 10^9^ /L), all four horses requiring early catheter removal (G46A, G46C, G15F, and G15I) developed an increase in the total leukocyte count, with neutrophilic leukocytosis at Day 7 (12.3 ± 2.2 × 10^9^/L; *p* = 0.036) and, all three horses (G46E, G15G, and G15H), at Day 15 as well (12.7 ± 1.9 × 10^9^ /L; *p* = 0.021). However, only the horses showing limb swelling and lameness 15 days after implantation (G46E, G15G, and G15H), developed an increase in serum fibrinogen in relation to the baseline values (4 ± 0.2 g/L to 7.3 ± 0.9 g/L; *p* = 0.011). Seven days after removing the catheters, the hematological exams returned to within the physiologic reference values for the species. Once the catheters were removed, the horses were observed to have reduced swelling, improved lameness and secondary intention wound healing without complications. No clinical signs of vascular impairment of the limb were detected 30 days after the catheters were removed, and ultrasonographic evaluation revealed reestablishment of the cephalic blood flow.

## Discussion

The present study is the first describing the adaptation of the surgical technique for the implantation of TICs in equine limbs. The use of TICs for repeated IVRLP in horses was not evaluated in this study. The TIC implantation technique was performed with a low level of difficulty, and the catheter allowed for IVRLP through the horse’s cephalic vein 24 h following placement. However, most of the catheters caused complications including limb swelling, pain, and lameness presumably secondary to catheter kinking and venous thrombosis, indicating that long-term use of this device may not be recommended in horses.

Excessive limb movement during IVRLP performed with a butterfly catheter or a short-term peripheral catheter can result in dislodgment of the catheter, swelling at the site of venipuncture, edema of the affected leg and lameness [17, 18]. Although TICs are more expensive in the short term, these devices have significant advantages. Their use in humans lowers the risk of infection, risk of catheter displacement and migration, and the cost of treatment in the long term [4, 15, 16]. For horses with bone or joint infections treated with IVRLP, the use of a TIC could eliminate the need for repeated venipunctures and possibly result in fewer complications and less discomfort for the patient, as suggested elsewhere [10, 12].

In this study, TIC implantation was technically uncomplicated. IVRLP was performed using contrast to illustrate the use of this venous device, and the contrasted radiographic images confirmed the absence of technical errors. However, the reservoir could have been placed a little more distally in the limb to facilitate the tourniquet placement. In this case, the cephalic vein would also need to be accessed more distally. The location of the limb to implant the reservoir is not a limiting factor of this technique because it is possible to create a subcutaneous tunnel between the site of the cephalic vein’s puncture and the reservoir, so that the catheter can be passed through it.

TICs placed in the cephalic vein of the horses studied herein lasted seven days in 40% of the horses (4/10), 15 days in 30% of the horses (3/10), and 60 days in 30% of the horses (3/10). Kelmer et al. [11] showed that 20G, 6.25 cm over-the-needle polyurethane catheters (Extended Use Catheter; Mila International) placed in cephalic, saphenous, and palmar digital veins of horses were fully maintained for six days and that 50% of the catheters placed in the cephalic vein survived for seven days. Also, the over-the-wire, 16G, 15 cm long, polyurethane catheter (OTW catheter; Mila International) is used routinely clinically for 1–2 weeks and, in specific cases, has been used for 3 weeks for IVRLP [12]. Comparatively, the TIC implanted in the cephalic vein of horses can remain functional as long as indwelling catheters, although the latter are easier to implant and cheaper. A disadvantage of the over-the-needle and over-the-wire catheters in relation to the TICs is that they are semi-implantable venous devices that need special care to protect their external portion against an accidental removal from the horse’s limb [11, 12]. In contrast, TICs are internal, warranting no concerns about their inadvertent removal after implantation.

Among the complications associated with TICs in humans, hematomas at the site of implantation, pneumothorax, hemomediastinum, and poor catheter positioning are classified as early stage complications [19] and infections or obstructions are the most frequent late-stage complications [6, 20–22]. In dogs, TIC has been used in oncologic patients, placed in the jugular vein with the reservoir implanted over the scapula. Infections in the area of the reservoir during the immediate postoperative, and late infections are the main complications reported [23]. In this study, two horses that received the long catheter (G46) and showed limb swelling and pain, lameness, and neutrophilic leukocytosis seven days after TIC implantation also presented catheter kinking in the region of the carpal joint. It was hypothesized that the kinking might have contributed to the vessel inflammatory stimulus and clinical signs. Similarly, five horses (one from G46 and four from G15) with limb swelling and pain, lameness, neutrophilic leukocytosis, and absence of blood flow in the catheterized region of the cephalic vein observed during ultrasonography, presented venous thrombosis, which is a complication often reported in human patients implanted with the TIC [20–22]. Venous thrombosis should be treated as a serious event that should be recognized early through rigorous clinical observation associated with rapid and sensitive diagnostic methods [24], as performed in this study.

The main cause of venous thrombosis is the lesion experienced by the endothelium during the manipulation of the catheter [21, 24]. In humans, the implantation of a central venous access causes the endothelium to lose its integrity and triggers the coagulation cascade by activating procoagulant factors and platelets and the consequent formation of thrombi [22]. Keeping the tip of the catheter close to or inside the right atrium, is a maneuver that contributes to reduce the occurrence of deep venous thrombosis associated with the catheter [6]. In this study, catheters cut to 46 cm in length presented less venous thrombosis cases (one horse) than catheters cut to 15 cm (four horses) in length. Keeping the tip of the catheter further from the point of cephalic vein puncture may have reduced the catheter movement inside the vessel during the horses’ locomotion and diminished the thrombogenic stimulus. Furthermore, to allow for repeated IVRLP, the catheter was inserted and maintained inside the cephalic vein in a retrograde direction to the blood flow, which probably favored the occurrence of endothelial injury and increased the pro-clotting stimulus [25]. This probably reduced the longevity of the totally implanted catheter as a functional venous access. Flushing the catheters with heparinized solution more frequently might also have increased the longevity of the TICs [11, 12].

Vein thrombosis with mural inflammation is the most common complication in horses with indwelling intravenous catheters [25, 26]. Thrombophlebitis may develop as a result of the physical presence of the catheter and/or contamination of the catheter by microorganisms. Bacterial contamination is thought to occur mainly as a result of migration of bacteria from the skin along the external surface of the catheter wall into the vein but may also be caused by a break in aseptic technique during catheter placement, administration of contaminated fluids, or contamination of the administration set [26]. In this study, aseptic technique was prioritized during catheter implantation and reservoir handling for IVRLP and patency tests. No local infection, either in the reservoir or in the catheter, was observed in any of the animals. Moreover, all skin sutures from the catheter implantation surgery healed by primary intention, and no exudate or tissue necrosis were observed at the time of the catheters’ removal. Nevertheless, culture the tip of the catheter searching bacteria, which could explain thrombophlebitis would have been very recommended and was not performed in this study. Monitoring of the catheters and the early diagnosis of complications, such as phlebitis or thrombophlebitis, could be improved if ultrasonography was performed daily. Early diagnosis should prompt the use of anti-inflammatory treatments and may increase the longevity of the catheters [11]. In this study, catheters were removed as soon as complications as limb swelling, pain, and lameness were noted.

The TIC enables insertion of 46 cm of catheter within the cephalic vein, thus reaching distal anatomical portions of the limb (middle third of metacarpus). However, multiple equine IVRLP pharmacokinetics studies reported that when cephalic vein is used, high therapeutic antibiotic concentration can be reached at the synovial structures of the distal limb (e.g-navicular bursa and distal interphalangeal joint) [17, 18, 27]. Thus, the long catheter may not be needed and instead could lead to complications as observed in two horses from the G46 that presented catheter kinking.

Considering that the TIC is easy to manage and has a high rate of satisfaction when used for central venous access in humans [15, 16, 22], studies that seek to make its use viable for the treatment of orthopedic infections, by means of repeated IVRLP, would benefit the welfare of the animals and the success of therapies, while minimizing pain and excessive handling of the horses. Future research is required to determine if TIC might be indicated to be implanted while the horse is anesthetized for surgical treatment of infected structures in the limb, and then used for repeated perfusions, while the horse is standing and sedated.

Some limitations should be noted in the present study. First, the small number of healthy horses used, and the results obtained make it difficult to consider the use of the TIC in clinical cases, mainly because of its limited longevity as a functional venous access. Second, daily investigation or repeated IVRLP through the totally implanted catheter were not performed. The present study only evaluated the implantation technique for TIC in horses, and the consequences of long-term implantation. Finally, the efficacy of TIC for use in IVRLP was not evaluated, and cannot be determined from this study.

## Conclusion

This study shows that the technique to implant the TIC in the cephalic vein of horses is feasible and can be performed with a low degree of difficulty. While the TIC may potentially reduce the number of venipunctures needed if used for IVRLP, the complications noted, such as limb swelling, lameness, and venous thrombosis were concerning. As the TIC failed to provide prolonged venous access for IVRLP in horses and demonstrated significant complications, use of this technique in clinical cases cannot be recommended.

## Materials and methods

### Adaptation of the technique for the implantation of the totally implantable catheter

Ten mixed breed adult horses participated in the study, including five geldings and five mares. The average age was 8 years (range: 4 to 12) and the average weight was 345 kg (range: 315 to 400 kg). Horses were clinically healthy according to physical evaluation, and all were sound when trotted. Horses were housed in separate stalls, had continuous access to fresh water, and were fed grass hay.

After six hours of fasting, the horses received xylazine (1 mg/kg bwt i.v.) as pre-anesthetic medication. Ten minutes later, the animals received guaiacol glyceryl ether (100 mg/kg bwt i.v.) for myorelaxation. Anesthetic induction was conducted with ketamine (2 mg/kg bwt i.v.) and midazolam (0.1 mg/kg bwt i.v.) mixed in the same syringe. Anesthesia was maintained with isoflurane and mechanical ventilation. Local infiltrative anesthesia with 10 mL of lidocaine hydrochloride was performed at the predetermined sites for catheter insertion and reservoir placement in their limbs. The same surgeon implanted all catheters.

**Fig. 7:**
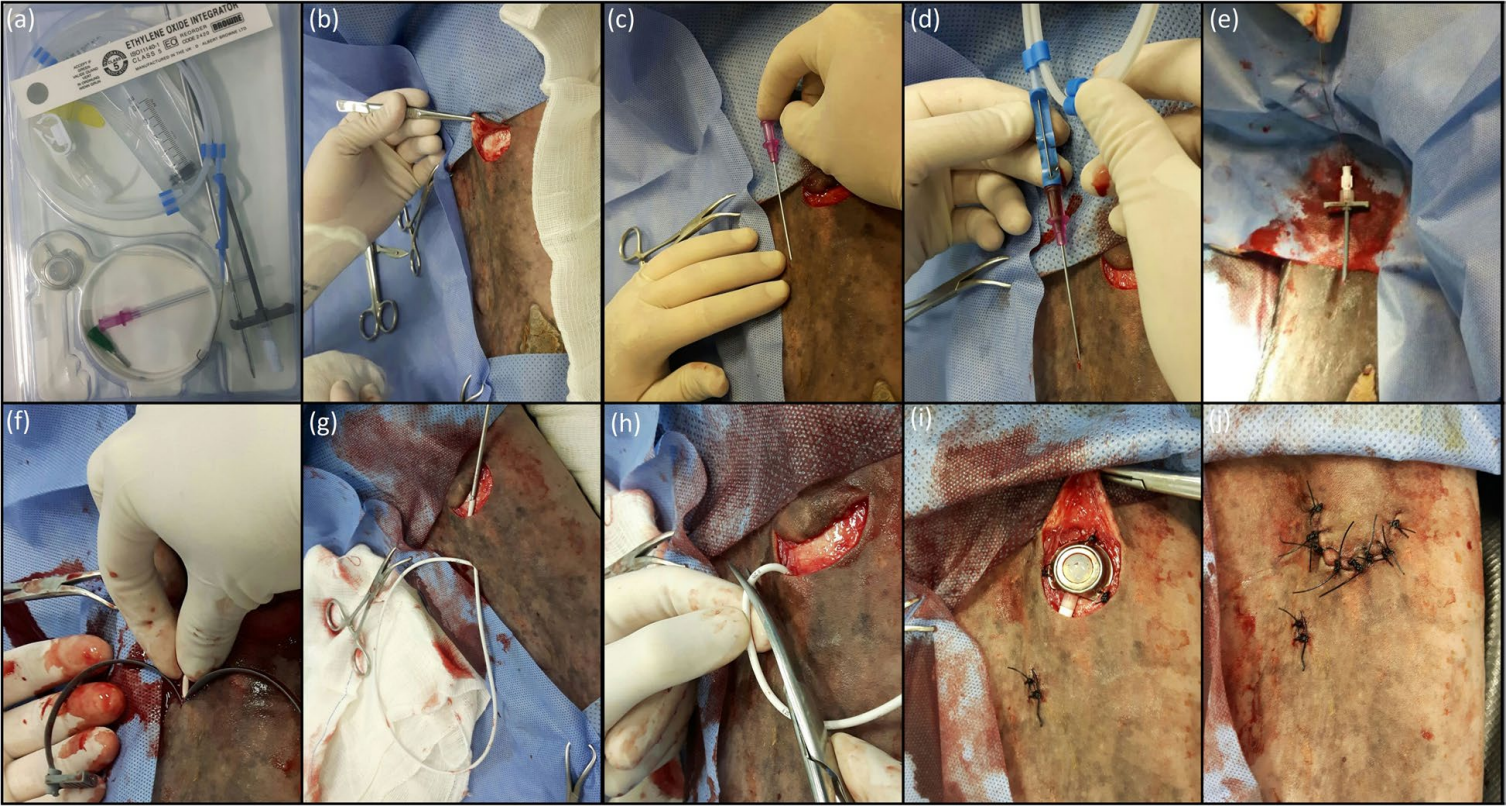
Images of the surgical implantation technique of the totally implantable catheter (**a**) in the cephalic vein of the right thoracic limb of an adult horse: (**b**) U-shaped skin incision, 10 cm, 3 cm caudal and 3 cm proximal to the determined point for puncture of the cephalic vein; (**c**) introduction of the puncture needle in the cephalic vein; (**d**) passage of the “J” shaped guide wire inside the puncture needle and the cephalic vein, followed by the removal of the puncture needle; (**e**) external portion of the guide wire positioned inside the introducer and dilator and their positioning inside the cephalic vein; (**f**) after positioning of the introducer and dilator inside the cephalic vein, removing of the guide wire and introducer, followed by the introduction of the totally implantable catheter and removal by “peel off” of the dilator; (**g**) catheter connected to the tunneling tool and passing through the subcutaneous tunnel created between the site of cephalic vein puncture and elliptical skin incision; (**h**) after radiographic evaluation, catheter excess being cut to be connect to the reservoir; (**i**) safe connection of the catheter to the reservoir, correct positioning and placement of the reservoir, suture of the reservoir to the muscle fascia inside the subcutaneous pocket in three points for suture anchorage, proper of the reservoir, with 2–0 nylon; note that the adaptation of the reservoir’s extremity to the catheter should be directed straight to the tunnel through which the catheter was passed, so as not to form curves and/or kinking in the connection between them; (**j**) suture of the skin over the reservoir and over the puncture point of the cephalic vein, with 0 nylon, simple interrupted suture pattern

The medial aspect of the antebrachium and the carpus of the right thoracic limb was clipped with a #40 clipper blade. Surgical preparation of the skin was in three stages: 4% chlorhexidine gluconate soap, scrubbed for 3 min, followed by isopropyl (70%) alcohol, and then chlorhexidine gluconate, 1:30 diluted with isopropyl alcohol (70%). As per the TIC (model Life-Port Titanium Infant 7.5 Fr; Neomex, Brazil) manufacturer’s instructions, the implantation procedure in the horses’ limbs involved performing a 10 cm U-shaped skin incision, 3 cm caudal and 3 cm proximal to the point defined for puncture of the cephalic vein, to create a subcutaneous pocket for reservoir implantation. Then, the silicone catheter was introduced into the cephalic vein, after perform a separate small skin incision prior to inserting the puncture needle, guide wire, introducer and “pell off” dilator into the vein, just proximal to the level of the chestnut. The silicone catheter was connected to a tunneling tool and passed through the subcutaneous tissues into the prior U-shaped incision. The reservoir was anchored within this incision to the underlying muscle fascia with 3, interrupted 2–0 nylon sutures. Then, the catheter was connected to the reservoir attempting to avoid curves and kinking. The skin was closed over the reservoir and over the puncture point of the cephalic vein with simple interrupted pattern suture using 0 nylon (Fig. 7).

Catheters cut to 46 cm in length were implanted into the right cephalic vein of five anesthetized horses (horses G46A, G46B, G46C, G46D, and G46E). These TICs extended to the middle third of the metacarpus, and the group was named collectively Group 46 (G46). Catheters cut to 15 cm in length were implanted into the right cephalic vein of five other anesthetized horses (horses G15F, G15G, G15H, G15I, and G15J). These TICs extended to the carpal canal, and the group was named collectively Group 15 (G15). The non-implanted contralateral limb constituted the control.

During the surgical procedure, radiographic images, centered at the mid-radius and carpus (G15) or at the mid-radius, carpus and mid-metacarpus (G46), were obtained in mediolateral, dorsolateral-palmaromedial, dorsopalmar, and dorsomedial-palmarolateral projections, to evaluate the catheter positioning and detect the presence of kinking. A Poskon emitter (PXP-20HF PLUS model) and DR Serv Image System (SIMS DR 35 model) were used to acquire the images.

### Post-implantation procedures for the totally implantable catheter

After assisted anesthetic recovery, the animals were housed in individual stalls. The skin incisions were cleaned once a day and local non-stick bandages were maintained over the sutures. The animals received three doses of 20,000 IU/kg bwt i.m. of benzathine benzylpenicillin every 48 h, and 4.4 mg/kg bwt i.v. of phenylbutazone every 24 h for 3 days. The skin sutures were removed 10 days postoperatively. Twenty-four hours after the implantation of the catheter, a second radiographic evaluation was performed to evaluate catheter location and alignment. Next, IVRLP was performed to evaluate the implant’s feasibility.

To properly handle the TIC, aseptic surgical preparation included spiral movements on the skin and over the reservoir with povidone-iodine followed by isopropyl (70%) alcohol. Subsequently, the region over the reservoir was massaged with an anesthetic ointment (Emla, Astrazeneca, Brazil) to reduce the discomfort of the skin puncture. To access the titanium reservoir, a sterile Huber-type needle (20G × 20 mm; Neomex, Brazil) was used to puncture the skin at a 90° angle and then cross the silicon septum until its tip touched the bottom of the reservoir. To avoid skin lesions over the reservoir and avoid complications such as infection, the reservoir was divided into four quadrants. The needle insertion site was rotated, and the quadrant used recorded. After each puncture, a bandage made with micropore adhesive was performed [28].

To perform the contrast IVRLP, rolled gauze was first applied over the cephalic vein and secured with bandage tape to attain better compression of the vascular structures. Then, a rubber tube tourniquet (1 m × 12 mm) was applied to the limb, just proximal to the reservoir implant, and tightened over the gauze pads. One hundred milliliters of low osmolarity contrast (Omnipaque® ioexol 300 mg I/ml) were injected by hand, at a 10 mL/min infusion rate, using a 60-mL syringe connected to an extension line coupled to the Huber needle. Immediately after the contrast administration, the catheters were flushed with 10 mL of heparinized saline **(**200 IU/mL). There was no exsanguination of the perfused region. After 30 min, the tourniquet was removed. Dispersion of the infused solution was observed by radiographic studies performed in mediolateral and dorsopalmar views, during and at 10 and 30 min after contrast infusion.

Patency tests of TICs were performed every 2 days after the implantation until day 15, and then weekly afterward. The patency test involved the collection of 5 mL of blood and subsequent infusion of 10 mL of 0.9% NaCl through the silicone septum of the reservoir to test the resistance of the extension line and detect possible leaks. The presence of venous return and/or easy and painless infusion of the solution represented a positive patency test [29]. They were described as flushable or non-flushable (obstructed) catheters. Subcutaneous infiltration was also identified when the reservoir region showed any increase in volume. Immediately after the patency tests, the flushable catheters were infused with 10 mL of heparinized saline **(**200 IU/mL).

Animals were examined twice a day for mucous membrane color, capillary refill time, heart and respiratory rates, intestinal motility, and rectal temperature. Lameness was evaluated daily at a walk, and the horses were assigned as either sound or lame. Hematological evaluation was performed at Day 0 (baseline; before implantation of the catheter), and at 7, 15, 30, and 60 days after implanting the catheter. It consisted of red blood cell count, packed cell volume, total protein, global and differential leukocyte counts, and plasma fibrinogen. The surgical wounds were evaluated daily by describing their gross appearance while assessing the animals for pain, swelling, heat, drainage, and skin healing following the removal of the sutures. Complications such as phlebitis and inflammation in the region near the site of catheterization were also evaluated.

Catheters were removed after 60 days from the horses showing no complications during the experimental period. The animals were sedated with xylazine (1 mg/kg; i.v.) followed by local infiltrative anesthesia with 10 mL of lidocaine hydrochloride. After clipping and aseptic preparation, a U-shaped skin incision was performed around the reservoir, and the catheter was removed from inside the cephalic vein. Then, the three sutures between the reservoir and muscle fascia were removed to release the reservoir. Finally, the skin was closed with simple interrupted suture pattern using 0 nylon. The skin incisions were cleaned daily and the sutures were removed after 10 days.

The catheters were removed immediately from horses that presented with limb swelling and lameness, and the wounds were left to heal by second intention. The animals were treated for possible infections with penicillin benzathine (20,000 IU/kg every 48 h, three doses), for pain with phenylbutazone (4.4 mg/kg, every 24 h, three days), and wound healing with iodine-based dressings. Prior to catheter removal, radiographic and/or ultrasound examinations were performed on animals whose catheters were not flushable or that developed swelling and lameness to investigate the underlying causes.

### Statistical analysis

A mixed statistical model was used to evaluate the normality and homogeneity of the experimental errors for clinical and hematological evaluations. The F test was used for significant effects. The between-times of the variables were analyzed through regression.

The survival of the catheters was tested for treatment differences by analysis of variance. The treatment factor included the length of the catheter. The effect of the catheters on lameness (yes/no or variable) was tested by using a logistic regression model with the same treatment factor. Individual treatment factors were also tested for their association with lameness by using Fisher’s exact test.

All analyses were performed with the MIXED procedure of the Statistical Analysis System (SAS, 1995) program, version 9.3. All the statistical tests used a significance level of *p* < 0.05.

## Data Availability

The datasets used and/or analyzed during the current study are available from the corresponding author on reasonable request.

## Abbreviations

IVRLP: Intravenous regional limb perfusion
TIC: Totally implantable catheter
